# Salubrinal Protects Against Cisplatin-Induced Cochlear Hair Cell Endoplasmic Reticulum Stress by Regulating Eukaryotic Translation Initiation Factor 2α Signalling

**DOI:** 10.3389/fnmol.2022.916458

**Published:** 2022-05-30

**Authors:** Wen Lu, Kun Ni, Zhuangzhuang Li, Lili Xiao, Yini Li, Yumeng Jiang, Jincheng Zhang, Haibo Shi

**Affiliations:** ^1^Department of Otolaryngology-Head and Neck Surgery, Shanghai Jiao Tong University Affiliated Sixth People’s Hospital, Shanghai, China; ^2^Department of Otolaryngology-Head and Neck Surgery, Shanghai Children’s Hospital, Shanghai Jiao Tong University, Shanghai, China; ^3^Department of Critical Care Medicine, Zhongshan Hospital, Fudan University, Shanghai, China

**Keywords:** cisplatin, endoplasmic reticulum stress, salubrinal, apoptosis, hearing loss, ototoxicity

## Abstract

**Objective:**

Cisplatin is a broad-spectrum anti-tumour drug commonly used in clinical practice. However, its ototoxicity greatly limits its clinical application, and no effective method is available to prevent this effect. Endoplasmic reticulum stress (ERS) is reportedly involved in cisplatin ototoxicity, but the exact mechanism remains unclear. Therefore, this study aimed to investigate the role of eukaryotic translation initiation factor 2α (eIF2α) signalling and its dephosphorylation inhibitor salubrinal in cisplatin ototoxicity.

**Methods:**

We evaluated whether salubrinal could protect against cisplatin-induced damage in House Ear Institute-Organ of Corti 1 (HEI-OC1) cells and mouse cochlear explants. By knocking down eIF2α, we elucidated the vital role of eIF2α in cisplatin-induced damage in HEI-OC1 cells. Whole-mount immunofluorescent staining and confocal microscopy of mouse cochlear explants and HEI-OC1 cells were performed to analyse cisplatin-induced damage in cochlear hair cells and the auditory cell line.

**Results:**

Data suggested salubrinal attenuated cisplatin-induced hair cell injury by inhibiting apoptosis. In addition, salubrinal significantly reduced ERS levels in hair cells *via* eIF2α signalling, while eIF2α knockdown inhibited the protective effect of salubrinal.

**Significance:**

Salubrinal and eIF2α signalling play a role in protecting against cisplatin-induced ototoxicity, and pharmacological inhibition of eIF2α-mediated ERS is a potential treatment for cisplatin-induced damage in the cochlea and HEI-OC1 cells.

## Introduction

Drug-induced hearing loss is one of the most common types of sensorineural hearing loss (Cunningham and Tucci, 2017). Cisplatin is a broad-spectrum anti-tumour drug commonly used in clinical practice, but its extensive and severe adverse effects on normal tissues greatly limit its clinical application (Wilson et al., 2017). Ototoxicity and nephrotoxicity are the main side effects of cisplatin (Gentilin et al., 2019). Diuretic hydration and other methods may prevent cisplatin nephrotoxic injury (Duan et al., 2020; Bégin et al., 2021). However, the FDA has not yet approved any drug that can effectively prevent cisplatin-induced hearing loss. According to the latest World Hearing Report, 23–50% of adults and up to 60% of children treated with cisplatin suffer from significant hearing loss (Chadha et al., 2021). Hearing loss has a negative effect on a person’s quality of life and creates a heavy healthcare burden (Knight et al., 2005; Frisina et al., 2016; Brock et al., 2018). To date, no proven strategy has been established for preventing and treating cisplatin-induced hearing damage, mainly because the mechanism of cisplatin-induced cochlear damage is not fully understood. It is acknowledged that cochlear hair cell (HC) and spiral ganglion cell death is a main pathology contributing to cisplatin-induced hearing loss (Kros and Steyger, 2019). However, an in-depth mechanism of cisplatin damage to cochlea is not well understood, highlighting the importance of further investigation.

The endoplasmic reticulum is found in eukaryotic cells, and it is directly associated with cholesterol metabolism, membrane/secreted proteins, phospholipids, calcium ion signals, and aminoglycosides (Luongo et al., 2017). When the body is subjected to unfavourable stimulation, such as ischemia-reperfusion injury, the endoplasmic reticulum lumen environment is destroyed due to a disorder in Ca^2+^ metabolism (Luongo et al., 2017). As a result, the newly synthesised protein cannot fold normally, resulting in an excessive protein load, which eventually triggers endoplasmic reticulum stress (ERS) (Oyadomari and Mori, 2004; Delaunay-Moisan and Appenzeller-Herzog, 2015; Hetz et al., 2020). An increasing number of studies found that ERS was involved in drug-induced hearing loss and played an important role in the development of ototoxicity, but the precise molecular mechanism was still unclear (Jia et al., 2018; Wen et al., 2021).

Salubrinal (Sal) is a selective protein phosphatase I complex inhibitor, which dephosphorylates eIF2α (Boyce et al., 2005; Binet and Sapieha, 2015). EIF2α phosphorylation reduces the expression of reaction initiators, resulting in reduced synthesis of unfolded proteins and the preservation of the ability to synthesise proteins, which is beneficial for ERS reduction (Hetz, 2012). Therefore, Sal can reduce ERS overexpression and block the initiation of the apoptotic program by selectively inhibiting the dephosphorylation of the PERK-eIF2α phosphate complex (Boyce et al., 2005). Sal plays a cytoprotective role during ERS in various diseases (López-Hernández et al., 2015; Matsuoka and Komoike, 2015; Font-Belmonte et al., 2019). Our previous studies revealed (Zhang et al., 2015, 2020) that Sal treatment improved neurological dysfunction through the inhibition of ER stress after postresuscitation brain injury. This finding suggests that ERS inhibition with Sal could be a neuroprotective strategy. However, whether activating eIF2α phosphorylation with Sal in cochlear HCs could inhibit ERS and thus protect against cisplatin-induced hearing loss is unknown.

In this study, we aimed to investigate the role of eIF2α signalling in cisplatin-induced cochlear damage and establish an effective therapy against cisplatin induced-hearing loss. We hypothesised that Sal could attenuate cisplatin-induced auditory HC apoptosis by inhibiting ERS and this effect was dependent on the eIF2α phosphorylation.

## Materials and Methods

### Animals

All animals were purchased from Shanghai Jie Si Jie Laboratory Animal Co., Ltd. (Shanghai, China). C57BL/6 mice were used in this study for the cochlear explant experiments. Total 28 mice aged P3 was used in our experiments. All experiments were conducted in accordance with the recommendations of the Constitution of the Animal Ethical and Welfare Committee. The study protocol was approved by the Animal Research Committee of the Shanghai Jiao Tong University Affiliated Sixth People’s Hospital. Efforts were made to minimise animal pain and the number used as much as possible.

### Materials

We used the following reagents and antibodies: Sal (HY-15486; MedChemExpress, Monmouth Junction, NJ, United States), cisplatin (P4394; Sigma-Aldrich, St. Louis, MO, United States), anti-BIP/GRP78 (5,174; Cell Signaling Technology, Danvers, MA, United States), anti-eIF2α (5,324; Cell Signaling Technology), anti-phospho-eIF2α-S51 (AP0692; ABclonal China, Wuhan, China), anti-cleaved caspase 3 (9,661; Cell Signaling Technology), anti-C/EBP homologous protein (CHOP) (2,895; Cell Signaling Technology), anti-cleaved PARP (9,544; Cell Signaling Technology), a rabbit anti-myosin-VIIa antibody (25–6,790; Proteus Bioscience, Ramona, CA, United States), DAPI (ab104139; Abcam, Cambridge, United Kingdom), anti-GAPDH (5,174; Cell Signalling Technology), anti-β-tubulin (2,128; Cell Signalling Technology), and Phalloidin-iFluor 488, 555 (ab176753, ab176756; Abcam), [anti-β-Actin(AC026; ABclonal China, Wuhan, China)].

### Cell Culture and Drug Administration

House Ear Institute-Organ of Corti 1 (HEI-OC1) cells provided by Dr. Iris Heredia were cultured according to the guidelines (Kalinec et al., 2016). Before use, the cell line was verified by performing an immunohistochemical analysis of myosin-VIIa, a marker of cochlear hair cells. HEI-OC1 cells were cultured in six-well plates and proliferated under non-permissive conditions (10% CO_2_, 33°C) in high-glucose Dulbecco’s Modified Eagle’s Medium (DMEM) containing 10% foetal bovine serum (Gibco; Thermo Fisher Scientific, Waltham, MA, United States) without antibiotics. When cells reached 80% confluence, subculture was performed using 0.25% trypsin/EDTA (Gibco). Cisplatin (P4394; Sigma-Aldrich) at a final concentration of 20 μM was added and incubated for 24 h to induce HEI-OC1 cell damage.

### Cochlear Explants and Drug Administration

The cochlear tissues of C57BL/6J mice on postnatal day 3 (P3) were quickly dissected and cleaned in cold phosphate-buffered saline (PBS) (Sangon Biotech Co., Ltd., Shanghai, China). Next, the cochlea was cultured on glass coverslips coated with Cell-Tak in four-well plates (BD Biosciences, Franklin Lakes, NJ, United States) with DMEM/F12 growth medium (11330-032; Gibco) containing B-27 supplement (17504-44; Gibco), N-2 supplement (17502-048; Gibco), and ampicillin (50 g/mL, A5354-10ML; Sangon Biotech Co., Ltd.) at 37°C in an incubator environment of 5% CO_2_ and 95% O_2_. After incubation for a day, we pretreated cochlear explants with or without Sal (10 μM) and then co-treated them with 20 μM cisplatin for 24 h. Cisplatin at a concentration of 20 μM was selected based on our previous study (Li et al., 2022). After removing the cisplatin-containing medium, the cochlear explants were recovered in normal medium for 36 h.

### Measurement of Cell Viability

Cell viability analyses were performed using a cell counting kit-8 assay (CCK-8; HY-K0301; MedChemExpress). We trypsinised cells with 0.25% trypsin/EDTA, centrifuged them at 1,000 rpm for 3 min, and resuspended them in medium. Thereafter, HEI-OC1 cells were seeded at a density of 4,000 cells/well in 96-well plates. After 24 h of incubation, the medium was replaced with medium containing cisplatin (20 μM) and different doses of Sal. After 24 h of incubation, CCK8 solution was added per well and incubated with the cells at 37°C for 1 h. Finally, we used a microplate reader to determine the absorbance at 450 nm. The above experiment was repeated thrice.

### Immunofluorescence

Immunofluorescence experiments were performed to determine the different apoptosis and ERS markers. After different treatments, the HEI-OC1 cells or cochlear explants were washed with PBS thrice for 15 min. Afterward, we fixed HEI-OC1 cells or cochlear explants with 4% paraformaldehyde (Sangon Biotech Co., Ltd.) for 1 h at 25^°^C and then washed them thrice with 0.01 M PBS (Sangon Biotech Co., Ltd.). After permeabilisation with 1% Triton X-100 (Solarbio Life Sciences, Beijing, China) for 30 min, non-specific sample binding sites were blocked with 5% bovine serum albumin in PBS (Sigma-Aldrich) at 25^°^C for 1 h and then incubated with primary antibodies at 4°C overnight (dilution: 1:200–1:500). Subsequently, the samples were washed thrice with PBS and incubated with secondary antibodies (A32723, A32731, A32727, and A32732; Thermo Fisher Scientific) at room temperature for 1 h. Thereafter, the auditory HCs were labelled with FITC-labelled phalloidin (P5282; Sigma-Aldrich), and cell nuclei were labelled with DAPI (Sigma-Aldrich). Finally, we washed the cells thrice with PBS, mounted them with DAPI, and imaged them using an LSM 710 confocal microscope (Zeiss, Oberkochen, Germany). The fluorescence intensity of CHOP was measured using Image J software (NIH, Bethesda, MD, United States).

### Western Blotting

HEI-OC1 cells were lysed and prepared in RIPA buffer (Sangon Biotech Co., Ltd.) containing protease and phosphatase inhibitor cocktail (Sangon Biotech Co., Ltd.) on ice for 30 min. Thereafter, cell lysates were centrifuged at 12,000 rpm for 20 min, and the supernatants were collected for protein quantification. Protein concentrations were quantified using a BCA protein assay kit (Sangon Biotech). Equal amounts of proteins were loaded on 10% sodium dodecyl sulphate-polyacrylamide gel for separation *via* electrophoresis and then transferred onto nitrocellulose membranes, which were then blocked with 5% skim milk powder in Tris-buffered saline containing 0.1% Tween 20 (TBST) (Sangon Biotech) for 1 h at room temperature. Subsequently, the membranes were incubated with anti-BIP (5,174; Cell Signaling Technology), anti-eIF2α (5,324; Cell Signaling Technology), anti-phospho-eIF2α-S51 (AP0692; ABclonal), anti-cleaved caspase 3 (9,661; Cell Signaling Technology), anti-CHOP (2,895; Cell Signaling Technology), anti-cleaved PARP (9,544; Cell Signaling Technology), anti-GAPDH (5,174; Cell Signaling Technology), and anti-β-tubulin (2,128; Cell Signaling Technology). The antibodies used ranged from 1:500 to 1:1,000. After incubation with the primary antibodies overnight at 4°C, the membranes were washed thrice for 30 min with 1 × TBST and then incubated with the corresponding secondary antibodies (AS014 and AS003; ABclonal) at room temperature for 2 h. The cell membranes were examined using an Omni-ECL Femto Light Chemiluminescence Kit (SQ201; Epizyme, Cambridge, MA, United States) and imaged using a ChemiDocXRS imaging system (Bio-Rad Laboratories, Hercules, CA, United States). ImageJ software was used to measure band intensities. Each experiment was repeated more than thrice.

### Small Interference RNA Transfection

We designed three eIF2α-specific mouse Small Interference RNA (siRNAs) (RiboBio, Guangzhou, China) to knock down eIF2α expression in HEI-OC1 cells. A siRNA encoding a nonsense sequence was designed as the negative control. HEI-OC1 cells were transfected with eIF2α-siRNA or negative-siRNA according to the manufacturer’s instructions. HEI-OC1 cells were plated in six-well culture plates at a density of 3 × 10^5^ and transfected with siRNA using Lipofectamine 3000 (L3000001; Invitrogen, Waltham, MA, United States) for 24 h. The transfection efficiency was measured using western blotting. The following siRNAs were used to knock down eIF2α expression: 5′-TCCAAGAGCTTGAAGATTT-3′, 5′-CCTTCTCCAAGAGCTTGAA-3′, and 5′-CTCCAAGAGCT TGAAGATT-3′.

### Cell Counts

For HC quantification, cells labelled with myosin7a and showing normal nuclei were considered surviving HCs. To quantify HCs, we imaged the entire cochlea using a Zeiss microscope with a 40 × lens and used ImageJ software to quantify immunopositive cells. The average number of HCs per 130 μm in the apical, middle, and basal turns of the cochlea was calculated for each group. For quantification of apoptotic HCs, we selected only the middle portion of each explant.

### Statistical Analysis

Data were analysed using GraphPad Prism statistical software (version 8, GraphPad Software, Inc., San Diego, CA, United States). Normally distributed measures are expressed as mean ± Standard Error of Mean (SEM). Independent sample *t*-tests were performed for comparisons between two groups. One-way ANOVA was used for comparisons between multiple groups. *P*-values < 0.05 were considered statistically significant.

## Results

### Sal Reduced the Cytotoxicity of Cisplatin-Induced House Ear Institute-Organ of Corti 1 Cells

**FIGURE 1:**
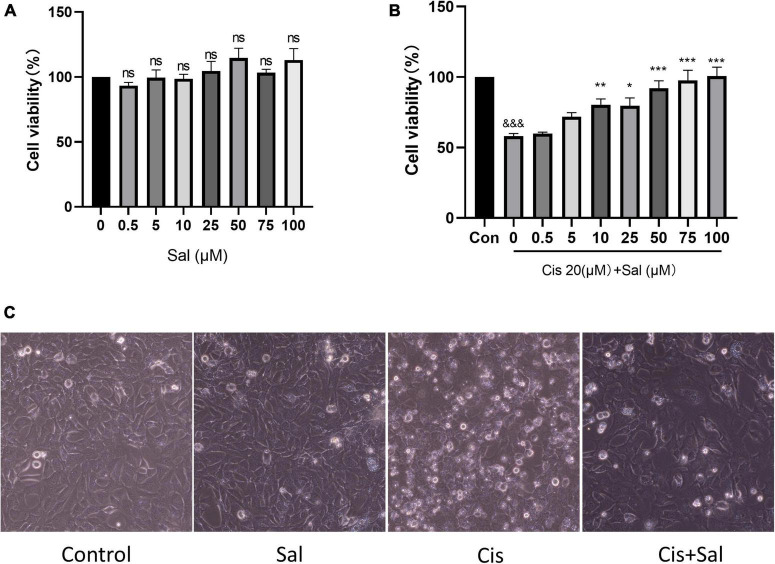
Sal enhances the viability of HEI-OC1 cells after cisplatin damage. **(A)** HEI-OC1 cells were treated with different Sal concentrations for 24 h. **(B)** HEI-OC1 cells were pretreated with or without different Sal concentrations (0–100 μM) for 4 h, followed by 20 μM co-treatment for 24 h except for those in the control group. **(C)** Image of HEI-OC1 cells cultured with different Sal or cisplatin concentrations. Scale bars = 100 μm. Cell viability was measured using the CCK-8 assay. Values are presented as mean ± SEM from independent experiments. *P < 0.05, **P < 0.01, and ***P < 0.001 vs. the control group; ^&&&^*P* < 0.001 vs. the cisplatin group. ns, no statistical difference; Sal, salubrinal; Cis, cisplatin; con, control.

Our previous study determined that cisplatin damaged HEI-OC1 cells appropriately at a concentration of 20 μm for 24 h (Li et al., 2022). HEI-OC1 cells were treated with different Sal concentrations (0–100 μM) for 24 h to determine its toxicity. The CCK-8 results showed that Sal did not decrease the viability of HEI-OC1 cells (Figure 1A). Therefore, HEI-OC1 cells were treated with or without Sal to determine whether Sal could protect against cisplatin-induced damage. The CCK-8 results showed that viability increased gradually with increasing doses of Sal (0–100 μM) (Figure 1B). Since the viability of 50 μM Sal-treated HEI-OC1 cells did not significantly differ from that of 100 μM Sal-treated cells following cisplatin exposure, 50 μM Sal was selected as the treatment condition for 24 h in the rest of this study. Moreover, dead cells on the top of the monolayer are more numerous in the cisplatin-treated group, while 50 μM Sal significantly alleviated this damage (Figure 1C). These findings suggest that Sal could effectively reverse cisplatin-induced ototoxicity at an appropriate dose.

### Sal Alleviated Cisplatin-Induced Apoptosis in House Ear Institute-Organ of Corti 1 Cells

**FIGURE 2:**
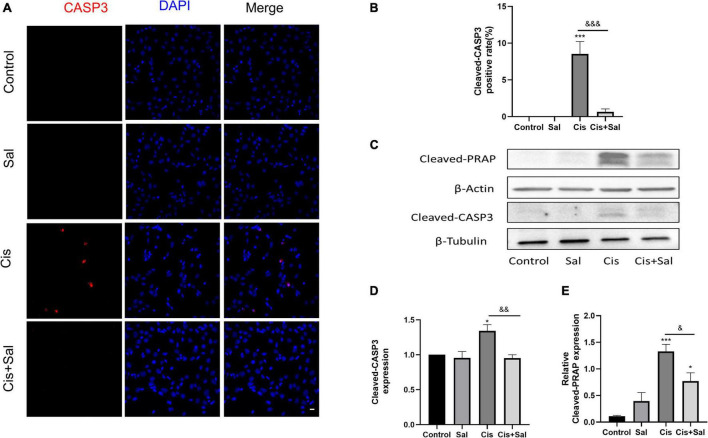
Sal reduces cisplatin-induced apoptosis in HEI-OC1 cells. **(A)** Cleaved caspase 3 (red) and DAPI (blue) double staining showing apoptotic cells after the different treatments (*n* = 3). **(B)** Quantitative analysis of apoptotic cells in **(A)**. **(C)** Western blotting results showing cleaved PARP (*n* = 3) and cleaved caspase 3 (*n* = 3) in the different groups. **(D,E)** Quantitative analysis of the western blotting results in **(C)**. Values are presented as mean ± SEM from independent experiments. *P < 0.05 and ***P < 0.001 vs. the control group; ^&^*P* < 0.05, ^&&^*P* < 0.01, and ^&&&^*P* < 0.001 vs. the cisplatin group. Sal, salubrinal; Cis, cisplatin. Scale bars = 20 μm.

To determine the protective effect of Sal in HEI-OC1 cells, we measured the percentage of apoptotic cells in the control, Sal, cisplatin, and cisplatin + Sal groups. Immunofluorescence analysis of cleaved caspase 3 showed that the number of cisplatin-induced apoptotic cells was reduced after Sal treatment (Figures 2A,B). Consistent with the immunofluorescence results, western blotting showed that the expression of cleaved caspase 3 and cleaved-PARP was significantly reduced after Sal treatment (Figures 2C–E). These findings suggested that Sal attenuated cisplatin-induced apoptosis in HEI-OC1 cells.

### Sal Decreased Cisplatin-Induced Apoptosis in Cochlear Hair Cells

**FIGURE 3:**
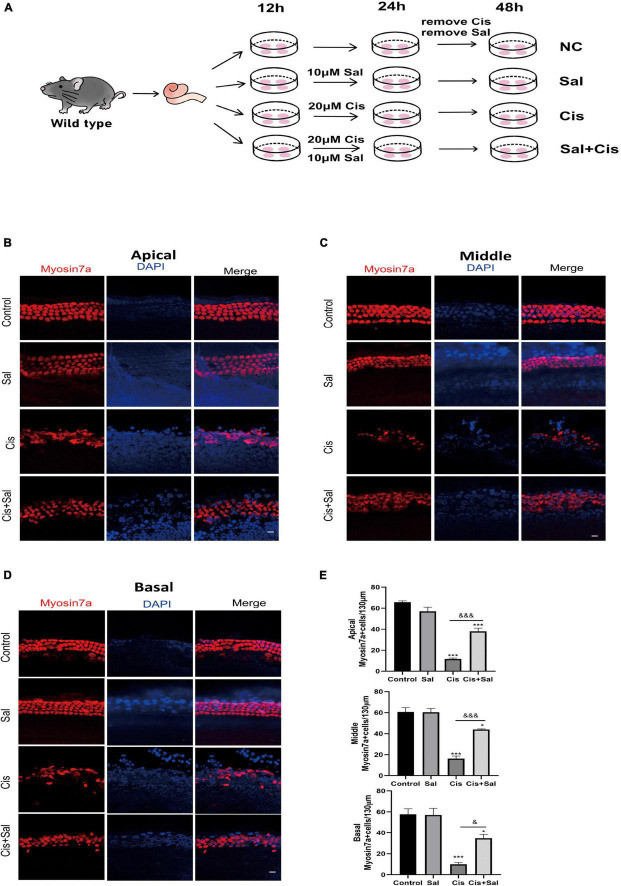
Sal promotes hair cell (HC) survival in the cochlea after cisplatin exposure. **(A)** Schematic diagram of drug addiction in tissue culture. **(B,D)** HCs in the apical **(B)**, middle **(C)**, and basal turns **(D)** of the cochlea were stained with anti-myosin-VIIa antibody and DAPI in the control, 10 μM Sal, 20 μM cisplatin, and 20 μM cisplatin + 10 μM Sal groups. **(E)** Quantification of the number of myosin VIIa-positive cells in the apical, middle, and basal turns of the cochlea. Values are presented as mean ± SEM from independent experiments. *P < 0.05 and ***P < 0.001 vs. the control group; ^&^*P* < 0.05 and ^&&&^*P* < 0.001 vs. the cisplatin group. Scale bars = 10 μm.

**FIGURE 4:**
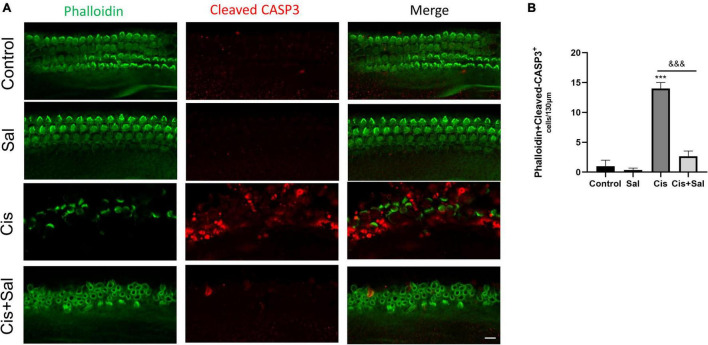
Cisplatin-induced hair cell apoptosis decreases after treatment with Sal. **(A)** Number of cleaved caspase-3-positive cells (red) decreased in the 20 μM cisplatin + 10 μM Sal group compared with that in the cisplatin group (middle turns). **(B)** Quantification of the number of phalloidin + cleaved caspase-3 double-positive cells in **(A)**. Values are presented as mean ± SEM from independent experiments. ***P < 0.001 vs. the control group; ^&&&^*P* < 0.001 vs. the cisplatin group. Scale bars = 10 μm.

To determine the effect of Sal in protecting cochlear HCs after cisplatin damage, we used cochlear organ explant cultures. The cochleae of C57BL/6 mice were dissected at P3, and the surrounding tissue and bone were removed using PBS. Cochlear explants were glued to glass cover slides coated with Cell-Tak (BD Biosciences) and divided into the following groups: control, Sal alone, cisplatin, and Sal + cisplatin (10 μM Sal pretreated for 4 h and 20 μM cisplatin co-treated for 24 h) (Figure 3A). To better characterise cochlear HCs, we performed immunofluorescence staining using antibodies against myosin-VIIa. Consistent with the results for HEI-OC1 cells, the number of HCs was significantly reduced in the cisplatin alone group, whereas Sal treatment significantly increased the number of HCs after cisplatin exposure (Figures 3B–E). Furthermore, no significant difference was found between the Sal alone and control groups, suggesting that Sal at 10 μM did not affect the survival of HCs. To further validate these results, immunofluorescence staining of cleaved caspase 3 was performed to analyse apoptotic HCs in different groups. Consistent with the number of HCs, the number of cleaved caspase 3-positive HCs was significantly increased in the cisplatin group, when10 μM Sal significantly reduced the cleaved caspase 3-positive HCs in the Sal + cisplatin group (Figure 4).

### Sal Attenuated Cisplatin-Induced Endoplasmic Reticulum Stress in Hair Cells and House Ear Institute-Organ of Corti 1 Cells

**FIGURE 5:**
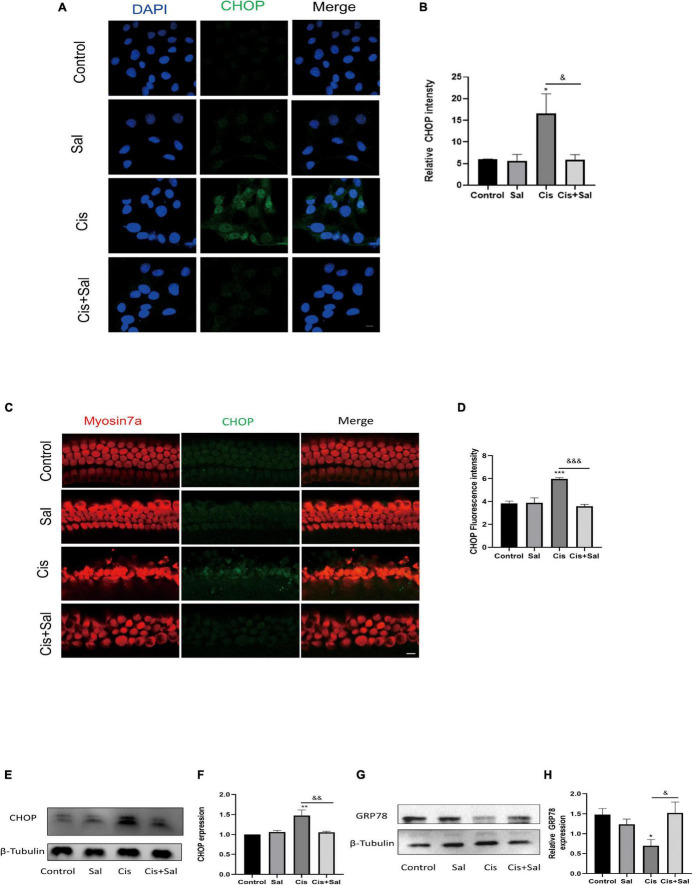
Sal attenuates cisplatin-induced endoplasmic reticulum stress in HEI-OC1 cells. **(A)** HEI-OC1 cells in the different groups were labelled with CHOP (*n* = 3). **(B)** Quantitative analysis of the data shown in **(A)**. **(C)** Cochlear basilar membranes in the different groups were labelled with CHOP (*n* = 3). **(D)** Quantitative analysis of the fluorescence intensity shown in **(C)**. **(E)** Western blotting results showing CHOP expression in HEI-OC1 cells after different treatments (*n* = 3). **(F)** Quantitative analysis of the data shown in **(E)**. **(G)** Western blotting results showing BIP expression in HEI-OC1 cells after different treatments (*n* = 3). **(H)** Quantitative analysis of the data shown in **(G)**. Values are presented as mean ± SEM from independent experiments. *P < 0.05, **P < 0.01, and ***P < 0.001 vs. the control group; ^&^*P* < 0.05, ^&&^*P* < 0.01, and ^&&&^*P* < 0.001 vs. the cisplatin group. Scale bars = 10 μm.

To determine whether Sal protects against cisplatin-induced damage by inhibiting ERS, the expression of CHOP and GRP78/BIP ERS markers was measured in HEI-OC1 cells in the different groups (Figure 5). Immunofluorescence staining showed that cisplatin treatment induced nuclear CHOP accumulation compared with the untreated controls (Figures 5A,B), whereas Sal and cisplatin co-treatment reduced CHOP expression and increased BIP expression. To further verify that Sal alleviated HC damage, we used immunofluorescence staining of CHOP in the cochlear explants. Similar to the results in HEI-OC1 cells, cisplatin treatment significantly increased CHOP expression, whereas Sal and cisplatin co-treatment significantly decreased CHOP expression (Figures 5C,D). Consistent with the immunofluorescence staining results, western blotting results showed that CHOP expression increased in the cisplatin group but decreased in the Sal group (Figures 5E–H). These data suggest that cisplatin caused significant ERS in cochlear HCs, and Sal could effectively reverse this damage.

### Sal Protected House Ear Institute-Organ of Corti 1 Cells Against Cisplatin-Induced Apoptosis by Eukaryotic Translation Initiation Factor 2α Regulation

**FIGURE 6:**
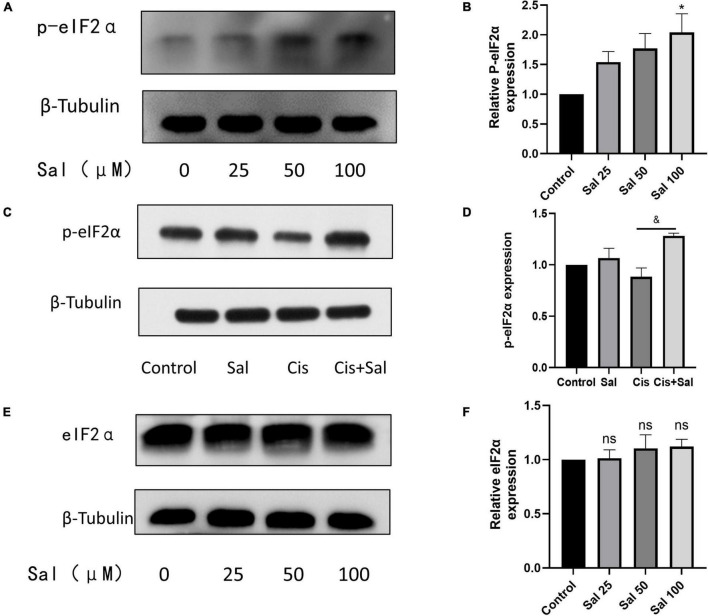
Sal inhibits cisplatin-induced hair cell endoplasmic reticulum stress by inhibiting the dephosphorylation of p-eIF2α. **(A)** As the concentration of Sal increases, p-eIF2α expression increases. **(B)** Quantitative analysis of the data shown in **(A)**. **(C)** Sal increases p-eIF2α expression in hair cell injury after cisplatin exposure. **(D)** Quantitative analysis of the data shown in **(C)**. **(E)** Western blotting results showing the expression of eIF2α after Sal treatment. **(F)** Quantitative analysis of the data shown in **(E)**. Values are presented as mean ± SEM from independent experiments. *P < 0.05 vs. the control group; ^&^*P* < 0.05 vs. the cisplatin group; ns, no statistical difference.

Sal selectively attenuates the dephosphorylation of phosphorylated eIF2α and protects cells from ERS-induced apoptosis (Zhang et al., 2015). p-eIF2α expression increased with increasing Sal concentration (Figures 6A,B). To investigate the role of p-eIF2α in cisplatin-induced cytotoxicity, we further analysed p-eIF2α expression after Sal treatment. Western blotting results showed that p-eIF2α expression significantly decreased in the cisplatin group but increased after Sal treatment (Figures 6C,D). Furthermore, Sal treatment did not affect the total amount of eIF2α expression (Figures 6E,F). These findings suggest that Sal protected HCs from cisplatin-induced apoptosis by activating the eIF2α pathway.

### Silencing Eukaryotic Translation Initiation Factor 2α Enhanced Cisplatin-Induced Hair Cell Death

**FIGURE 7:**
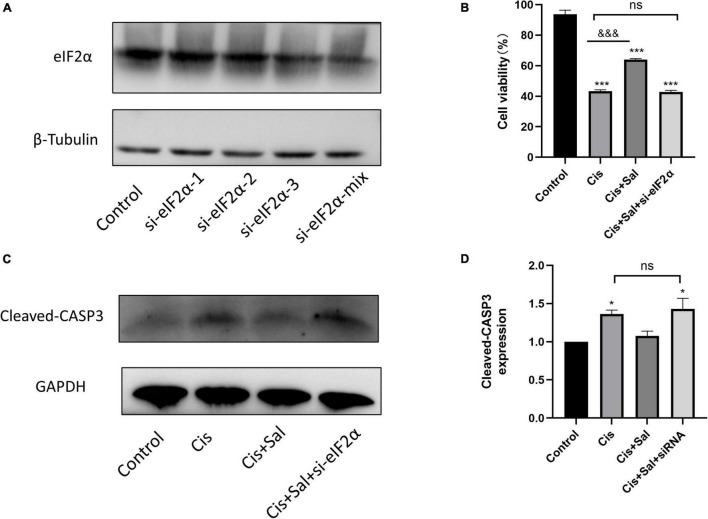
The protective effect of Sal was attributed to eIF2α in HEI-OC1 cells. **(A)** Western blotting results showing the expression of eIF2α transfected with eIF2α-siRNA. **(B)** Effect after eIF2α silencing on cisplatin-induced injury with or without Sal viability in HEI-OC1 cells. **(C,D)** Effects of knocking down eIF2α on cisplatin-induced apoptosis. Western blotting results showing the expression of cleaved caspase in the presence of cisplatin or Sal treatment after eIF2α silencing (*n* = 3). Values are presented as mean ± SEM from three independent experiments. *P < 0.05 and ***P < 0.001 vs. the control group; ^&&&^*P* < 0.001 vs. the cisplatin group. ns, no statistical difference; Sal, salubrinal; Cis, cisplatin.

We transfected HEI-OC1 cells with eIF2α-specific or control siRNA to determine whether eIF2α plays a direct role in the Sal-mediated protective effects against cisplatin-induced cell death. The best knockdown effect was observed in HEI-OC1 cells with a Si-eIF2α mixture (Figure 7A). After 36-h transfection, cell viability was analysed using the CCK-8 assay, and results showed that Sal significantly enhanced HEI-OC1 cell viability in the presence of cisplatin. However, cell viability was significantly reduced after eIF2α-specific siRNA treatment compared with that in the control siRNA-treated cells (Figure 7B). Furthermore, consistent with the CCK-8 results, western blotting results showed that the expression of cleaved caspase-3 was significantly increased in cells treated with eIF2α-specific siRNA (Figures 7C,D). These results suggest that eIF2α was responsible for the protective effects of Sal against cisplatin-induced apoptosis in HCs.

**FIGURE 8:**
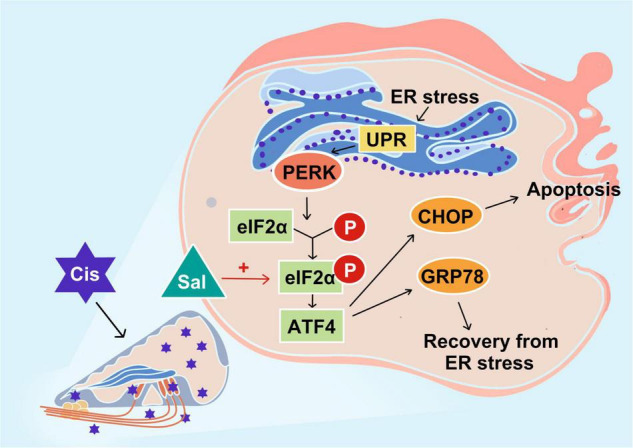
Schematic diagram showing the specific regulatory role of endoplasmic reticulum stress in cisplatin-induced ototoxicity and the main signalling branch exerting a pro-apoptotic effect. PERK and its related PERK/eIF2α/CHOP signalling branch are speculated to play a major role in cisplatin-induced ototoxicity. Sal increases the anti-apoptotic effect of GRP78 by inhibiting the dephosphorylation of eIF2α.

The results for both HEI-OC1 cells and cochlear explants showed that the PERK/ATF4/CHOP signalling branch plays an important role in cisplatin-induced ototoxicity (Figure 8).

## Discussion

Sal attenuated cisplatin-induced HC damage by inhibiting apoptosis. Furthermore, the effect of Sal was attributed to ERS inhibition, which was dependent on p-eIF2α activity. Knockdown of eIF2α reversed the protective effect of Sal against cisplatin-induced injury in HEI-OC1 cells, leading to cisplatin-induced apoptosis. These results demonstrate that cisplatin-induced HC apoptosis was associated with eIF2α signalling, one of the three major pathways of the unfolded protein response (UPR), while Sal could protect against cisplatin-induced ototoxicity in cochlear HC ERS.

Ototoxic chemicals including cisplatin, acetaminophen, and aminoglycoside disrupt the correct folding of protein or induce mistranslation, leading to the aggregation of unfolding or misfolding proteins in the ER of cochleae (Kalinec et al., 2014; Oishi et al., 2015; Qu et al., 2022). Once the excessive accumulation of unfolding or misfolding proteins occurs in the ER, severe ERS results in an inability to maintain ER homeostasis, leading to cell apoptosis and death (Hetz and Papa, 2018). ERS, evoked by misfolding and unfolding proteins in the ER, can activate the UPR to reduce ER workload by increasing the protein folding capacity, removing misfolded/unfolded proteins from the ER, and inhibiting translation to block new protein synthesis (Wang et al., 2020). A previous study showed that by inhibiting the accumulation of misfolded/unfolded proteins, upstream of ERS, could effectively attenuate cisplatin-induced damage in auditory HCs (Wen et al., 2021). However, the role of UPR downstream signalling has not been elucidated in HCs. Therefore, whether activating UPR could prevent cisplatin-induced ototoxicity should be further investigated.

UPR mainly consists of three distinct signalling pathways, namely protein kinase R-like endoplasmic reticulum kinase (PERK), activating transcription factor 6 (ATF6), and inositol-requiring kinase 1 (IRE1) (Nakka et al., 2016; Ren et al., 2021). Dysfunction and sustained changes in endoplasmic reticulum function lead to the release of GRP78 PERK, ATF6, and IRE1, which in turn activate associated downstream signalling molecules such as the ERS marker CHOP, ultimately leading to cell death through processes such as apoptosis (Hetz, 2012; Hu et al., 2018). In the current study, we focused on PERK-eIF2α signalling and further investigated the role of eIF2α in cisplatin-induced ototoxicity. The PERK-eIF2α signalling pathway is one of the most critical pathways for the survival of cells exposed to various stressors such as toxic environments, malnutrition, and oxidative stress (Rozpedek et al., 2016). Under ERS, GRP78 is dissociated, and then PERK is activated, which further promotes eIF2α phosphorylation. Phosphorylated eIF2α inhibits general translation, thereby reducing the protein burden of the endoplasmic reticulum and protecting cells from ERS (Godin et al., 2016). Sal has been identified as an inhibitor of eIF2α dephosphorylation (Boyce et al., 2005). Similar to these studies, our results showed that increasing concentrations of Sal increased p-eIF2α expression in HEI-OC1 cells, suggesting that Sal inhibited eIF2α dephosphorylation in a dose-dependent manner in auditory HCs. Furthermore, p-eIF2α decreased after cisplatin treatment, but this reduction was inhibited by Sal treatment, suggesting that p-eIF2α is crucial in cisplatin-induced ERS and apoptosis. However, the role of eIF2α is controversial. Although numerous studies support the notion that phosphorylated eIF2α is beneficial in ERS-induced cell death, eIF2α dephosphorylation has also been shown to contribute to cell survival (Novoa et al., 2003; Duan et al., 2014). Our data demonstrated that cisplatin-induced apoptotic cell death was attenuated if eIF2α could be selectively activated, whereas eIF2α knockdown enhanced cisplatin-induced apoptosis. These results further verified our view that the PERK-eIF2α pathway has a protective role in cisplatin-induced ERS in cochlear cells.

ATF4, which is downstream of eIF2α, has dual effects in ERS, including promoting the transcription of ER chaperones such as GRP78 to help ER recovery and increasing CHOP expression to induce apoptosis. CHOP, also known as growth arrest and DNA damage-inducible gene 153, is commonly expressed at very low levels in normal cells (Oyadomari and Mori, 2004). In response to various ERS stimuli, CHOP expression is upregulated and helps regulate cellular redox and cell death. Thus, CHOP upregulation is also a good biomarker of ERS and is a major downstream effector of the PERK pathway, which functions as a pro-apoptotic factor (Hu et al., 2018; Zhao et al., 2021). BIP plays a crucial role in regulating the dynamic homeostasis of the ER and is a sensor of ERS (Elfiky et al., 2020). BIP dissociates from PERK, IRE1, and ATF6 in response to ERS and triggers UPR, which possesses anti-apoptotic and cytoprotective effects (Hetz, 2012). In the current study, BIP expression decreased in the cisplatin-treated group but increased after Sal treatment. Contrastingly, CHOP was upregulated after cisplatin exposure when Sal strongly decreased the expression. Notably, upregulation of BIP/GRP78 is protective against ischemic injury and also against cisplatin-induced damage in HEI-OC1 cells (Ouyang et al., 2011; López-Hernández et al., 2015; Yi et al., 2020). Our results supported this point and further showed that pharmacological activation of eIF2α-BIP could alleviate cisplatin-induced ERS and apoptosis. Moreover, Sal significantly reduced CHOP expression in cisplatin-induced injury in HEI-OC1 cells and cochleae, suggesting that Sal could also regulate the eIF2α-CHOP pathway to inhibit cisplatin-induced ERS in HCs.

Apoptosis is a positively regulated form of cell death that is mainly involved in cisplatin-induced ototoxicity (Ruhl et al., 2019; Tang et al., 2021). Accumulating evidence indicates that inhibiting apoptosis could effectively protect against cisplatin-induced damage (Wang et al., 2004; Ruhl et al., 2019; Wu et al., 2020). Sal treatment has been proposed to prevent apoptosis in various disease models (López-Hernández et al., 2015; Li et al., 2019, 2020). Consistent with these studies, Sal significantly alleviated cisplatin-induced apoptosis in HEI-OC1 cells and HCs, as evidenced by PARP and caspase-3 levels. In addition, ERS has also been proposed as a potential mechanism for cisplatin-induced ototoxicity. An increasing number of studies have found that ERS plays an important role in drug-induced hearing loss (Gentilin et al., 2019). A previous study found that ERS is involved in spiral ganglion neuron apoptosis after chronic kanamycin-induced deafness (Tu et al., 2019). Moreover, taurine deoxycholic acid attenuates gentamicin-induced cochlear HC death *in vitro* by inhibiting ERS (Hu et al., 2018). In kidney and cancer cell tissues, ERS-induced apoptosis is one of the mechanisms of cisplatin-induced cytotoxicity (Huang et al., 2020; Tham et al., 2020). Our findings are consistent with previous studies showing that Sal effectively inhibits ERS levels, thereby preventing cisplatin-induced apoptosis in HEI-OC1 cells and cochlear HCs. Our results suggest that ERS was hyperactivated in HEI-OC1 cells and HCs after cisplatin exposure, which led to HC apoptosis, and inhibition of ERS could be a promising approach to reduce cisplatin-induced apoptosis. Taken together, our findings suggest that Sal reduces cisplatin-induced apoptosis in HEI-OC1 cells and HCs by attenuating ERS.

This study has several limitations. First, although Sal demonstrates effects associated with preventing cisplatin-induced ototoxicity, it has not been approved by the FDA for clinical application. Second, we demonstrated a protective effect of Sal in cochlear explants and HEI-OC1 cells *in vitro*, but its role *in vivo* was not further explored. Therefore, the role of Sal in protecting against cochlear cisplatin damage and the mechanism involved need to be further investigated and validated in animal models.

## Conclusion

In conclusion, our findings provide *in vitro* evidence that Sal significantly attenuates ERS in HEI-OC1 and cochlear HCs, subsequently suppressing cisplatin-induced apoptosis. In addition, eIF2α may be an important target for preventing and treating cisplatin ototoxicity. Furthermore, eIF2α plays a crucial role in ERS in HCs, and pharmacological enhancement of eIF2α phosphorylation is a potential therapeutic strategy for cisplatin-induced hearing loss.

## Data Availability Statement

The raw data supporting the conclusions of this article will be made available by the authors, without undue reservation.

## Ethics Statement

The animal study was reviewed and approved by the Ethics Committee of Sixth People’s Hospital Affiliated with the Shanghai Jiao Tong University.

## Author Contributions

WL, KN, and ZL performed all the experiments, analysed the data, and wrote the manuscript. JZ and HS conceived and designed all the experiments. WL and ZL acquired and analysed some of the data. LX, YL, and YJ helped with the experimental design and data interpretation. All authors contributed to the article and approved the submitted version.

## Conflict of Interest

The authors declare that the research was conducted in the absence of any commercial or financial relationships that could be construed as a potential conflict of interest.

## Publisher’s Note

All claims expressed in this article are solely those of the authors and do not necessarily represent those of their affiliated organizations, or those of the publisher, the editors and the reviewers. Any product that may be evaluated in this article, or claim that may be made by its manufacturer, is not guaranteed or endorsed by the publisher.
